# SELF-CONFIDENCE ON ACQUIRED SURGICAL SKILLS TO DEAL WITH SEVERE TRAUMA PATIENTS IN RECENTLY GRADUATED SURGEONS

**DOI:** 10.1590/0102-672020210001e1561

**Published:** 2021-05-14

**Authors:** Javier VELA, Leonardo CÁRCAMO, Caterina Contreras, Claudia ARENAS, Juan Pablo RAMOS, Rolando REBOLLEDO, Julián VARAS, Jorge MARTÍNEZ, Nicolas JARUFE, Pablo ACHURRA

**Affiliations:** 1 Pontificia Universidad Católica de Chile, Digestive Surgery, Santiago, Región Metropolitana, Chile; 2 Pontificia Universidad Católica de Chile, Simulation and Experimental Surgery Center, Santiago, Región Metropolitana, Chile; 3 Complejo Asistencial Dr. Sótero del Río, Unidad de Trauma y Urgencias, Santiago, Región Metropolitana, Chile; 4 Pontificia Universidad Católica de Chile, Intituto de Ingenería Biológica y Médica, Santiago, Región Metropolitana, Chile

**Keywords:** General surgery, Internship and residency, Clinical competence, Advanced Trauma Life Support Care, Wounds and injuries, Ferimentos e lesões, Internato e residência, Cirurgia geral, Competência clínica

## Abstract

**Background:**

Trauma is one of the leading causes of death in the world and proper surgical care is critical to impact mortality. In Chile, trauma associated death ranks first as mortality cause in population between 20 and 59 years old. Appropriate surgical skills are required to deal with these complex patients. Self-confidence to practice trauma procedures after the General Surgery Residency have not been reported in our country.

**Aim:**

Describe the level of self-confidence to deal with trauma procedures of surgeons who recently graduated from a General Surgery Residency.

**Method:**

Descriptive cross-sectional study. We designed and applied a survey in 2015, 2016 and 2017 to recently graduated surgeons, to inquire about self-confidence of surgical skills to deal with trauma scenarios. Eighteen trauma surgery procedures (including cervical, thoracic, abdominal and vascular procedures) were evaluated using a 5-grade Likert scale. The number of procedures performed during the residency was also queried.

**Results:**

Eighty-eight recently graduated surgeons from 11 different training programs in Chile were included. The report of competencies was high in procedures such as intestinal injuries, were 98% felt competent or very competent in their repair. On the other hand, in complex traumas such as major vessel injury, up to 76% reported not being competent. Self-confidence on procedures was directly associated with the number of procedures performed during residency.

**Conclusions:**

Recently graduated surgeons from General Surgery Programs report high levels of confidence to deal with low and intermediate complexity traumas, but a lower level of confidence to treat high complexity cases.

## Graphical abstract

[Fig f1a]

**Figure f1a:**
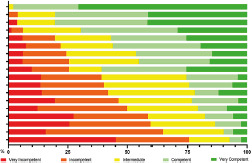
Level of competence by procedure

[Fig f1as]

**Figure f1as:**
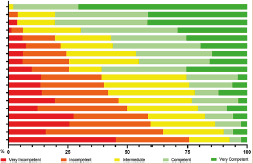
Nível de competência por procedimento

## RESUMO

**Racional:**

Trauma é uma das principais causas de morte no mundo e cuidados cirúrgicos adequados são críticos para determinar a mortalidade. No Chile, morte associada a trauma é a primeira causa de mortalidade na população entre 20 e 59 anos. Para lidar com esses pacientes complexos, são necessárias habilidades cirúrgicas precisas para esses procedimentos. Autoconfiança de cirurgiões recentemente graduados na Residência em Cirurgia Geral para práticas de procedimentos de trauma no nosso país não tem sido reportada.

**Objetivo:**

Descrever nível de autoconfiança para lidar com procedimentos de trauma em cirurgiões recentemente graduados na residência de cirurgia geral.

**Método:**

Estudo transversal. Foi desenhada e aplicada uma enquete em 2015, 2016 e 2017 a cirurgiões recentemente graduados para pesquisar sobre autoconfiança e habilidades cirúrgicas para lidar com cenários de trauma. Foram avaliados 18 procedimentos cirúrgicos de trauma (incluindo procedimentos cervicais, torácicos, abdominais e vasculares) usando a 5-grade Likert Scale. O número total de procedimentos feitos durante a residência foi avaliado.

**Resultados:**

Foram incluídos 88 cirurgiões recentemente graduados. O nível de competência foi reportado como alto em procedimentos como trauma intestinal, onde 98% sentiu-se competente ou muito competente em sua reparação. Por outro lado, em traumas complexos como dano vascular maior, até 76% reportaram não se sentirem competentes. A autoconfiança nos procedimentos esteve diretamente associada com o número de procedimentos realizados.

**Conclusões:**

Cirurgiões recentemente graduados na residência de cirurgia geral reportam níveis altos de confiança para lidar com traumas de complexidade baixa e média, mas um nível menor de confiança para tratar casos de complexidade alta.

## INTRODUCTION

During 2013 a total of 55 million people died worldwide, being four million (8,7%) caused by trauma^26^. In Chile, trauma is the fourth leading cause of death, corresponding to 7,5% of 2016’s deceases^16^. In the same year and concerning patients between 20 and 59 years old, the number of deaths associated with a road accident and assault (1.889) exceeds other common diseases such as ischemic cardiovascular disease (1.477) and hepatic cirrhosis (1.881)^7^. According to this data, trauma became the first cause of mortality in young patients with the consequent highest potentially productive years of life lost^2^. Seemingly, reports in Brazil identify that the most affected population are males and that the specific causes of trauma are falls and traffic accidents with an increasing rate due to motorbike accidents in the last years^20^ ^,^ ^36^. These numbers resemble our local reality were the reports identify traffic accidents and penetrating injuries as the specific leading causes of trauma^23^.

Mortality in the first hours of trauma is mainly attributed to bleeding of major vessels or highly perfused organs. Many of these deaths are avoidable with an early treatment where prehospital and hospital teams play a major role^5,33,35, 39^. Regarding the surgical treatment, the surgeon’s skills in the fast control of bleeding represent the critical step in reducing mistakes and, therefore, deaths. This approach is the primary focus of big trauma centers protocols^11^ ^,^ ^29^. The appropriate surgical approach also reduces the number of disabilities by limbs loss, the in-hospital days, and the use of resources associated with the care of these patients^3^ ^,^ ^12^.

In countries and locations where no trauma centers are available, the assessment and treatment of severe trauma are conducted by a general surgeon in the emergency department^14^. In this setting, support by specialist is not often available, and the surgeon must perform alone^4^. Moreover, most of these cases concentrate outside of working hours, making it even harder to obtain advice from a senior collegues^25^ ^,^ ^40^.

The training of general surgeons during the residency has changed in the last years^18^. Reduced working schedules, increasing safety concerns of the healthcare system, and an essential shift towards conservative management of trauma and other factors have resulted in a decreased exposure of residents to complex surgical cases^10^ ^,^ ^12^ ^,^ ^32^. In countries where the general surgery residency lasts for only three years, such as Chile, the factors mentioned above may have a more considerable impact than in more extended programs^4^. This situation has led to questioning the adequate competence to perform some procedures by the general surgeons with autonomy at the end of their training program^22^. Competence can be understood as a dynamic combination of knowledge, skills, and attitude necessary to perform a task efficiently. The overall measure of these factors is fundamental to assess the quality of any educational program. Nonetheless, there are no studies that assess the competence of surgeons in the treatment of severe trauma after graduating. 

Therefore, this study aims to describe the level of self-confidence to deal with trauma procedures of surgeons who recently graduated from a General Surgery Residency.

## METHODS

This study was approved by the institutional ethics committee with the number 170318009

### Assessment tools

**FIGURE 1:**
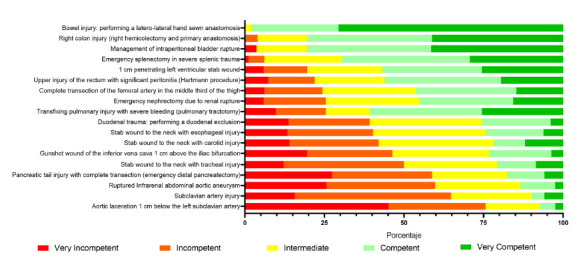
Level of competence by procedure. The percentage of each level of competence for each procedure is presented according to the self-reports

**FIGURE 2:**
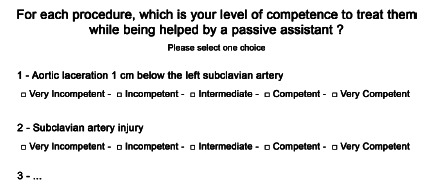
Example of the survey applied. This format was applied to all 18 trauma surgical procedures

A descriptive cross-sectional study was conducted. A self-assessment survey was designed and applied. The first section contained Likert type questions related to 18 trauma surgical procedures from cervical, vascular, thoracic, and abdominal areas (Figure 1)^21^. Participants were asked to report their assessment on their degree of competence (very incompetent - incompetent - intermediate- competent - very competent) to perform these trauma procedures being assisted by a first-year resident (an example can be seen in Figure 2). The listed procedures were selected according to frequent trauma cases and in line with the recommendation from the Chilean Surgical Society of minimum surgical skills required by a general surgeon^14^ ^,^ ^31^. Traumatic brain injury and orthopedic trauma were excluded because neither is managed by the general surgeons. The second section assessed the average number of surgical procedures performed during residency in seven trauma scenarios (Table 1).

**TABLE 1:** Number of procedures performed during the residency

APPROACH					
None	Between 1 y 5	Between 5 y 10	Between 10 y 20	20 or more	20 ou mais
Emergency Lapartomy for Abdominal Trauma	0%	18%	20%	23%	39%
Emergency Toracotomy for Thoracic Trauma	25%	43%	22%	10%	0%
CARDIO-THORACIC PROCEDURES					
	None	1 o 2	Between 3 y 5	Between 5 y 10	10 or more
Emergency Pulmonary Suture for Thoracic Trauma	45%	37%	11%	5%	2%
Emergency Cardiac Suture for Thoracic Trauma	47%	33%	18%	2%	0%
VASCULARES PROCEDURES					
	None	1 o 2	Between 3 y 5	Between 5 y 10	10 or more
Aorta Suture	79%	17%	4%	0%	0%
Arterio-Venous Fistula	40%	29%	21%	6%	4%
Vascular Bypass	33%	25%	28%	8%	6%

The survey was delivered via e-mail to 115 recently graduated general surgeons from all universities across the country between the years 2015 and 2017. The survey was sent yearly and answered between 1-3 months after graduation. 

### Statistical analysis

A descriptive statistical analysis was performed using RStudio^®^ (2019 v1.2.5001 on base R, Boston, US).

## RESULTS

A total of 88 recently graduated surgeons (71%) from 11 different programs answered the survey. From these, 31 graduated on 2015, 29 on 2016, and 28 on 2017. Figure 1 summarizes the results from the first section of the survey, and Table 1, those from the second section.

Cervical trauma concentrates the lowest levels of competence. In that anatomical segment, less than 25% report an acceptable level of competence to treat injuries of the trachea, esophagus, or carotid artery. In contrast, in thoracic trauma, the level of competence reported was intermediate, with 60% of participants stating an acceptable level of competence for the thoracic and pulmonary trauma. In abdominal trauma, the level of competence reported was high in most procedures such as bowel and colonic repair, intraperitoneal bladder rupture, and splenectomy, where more than 70% of the surgeons reported a high level of competence. The previous observation coincides with a high number of laparotomies performed during the residency (Table 1). At last, in complex limb trauma, the level of competence was intermediate and represented mainly by the trauma of the superficial femoral artery.

Specialty analysis shows that the lowest levels of competence are concentrated in vascular procedures, especially in those involving injuries of the subclavian artery, the vena cava, or the aorta. In contrast, the gastrointestinal procedures are the ones that report the highest levels of competence, especially the more common ones such as the injuries of the bowel, colon, and spleen. Nonetheless, there are exceptions, such as the trauma of the tail of the pancreas and duodenum, where the degree of competence was lower than 25%.

Table 1 summarizes the number of trauma procedures performed by recently graduated surgeons during their residency period. A critical number of participants performed a high number of emergency laparotomies (82% performed five or more), and only a small number rarely participated in this procedure (18% did between 0 and five laparotomies). In the thoracic area, we observed that 75% completed at least one emergency thoracotomy. However, only 20 % performed more than two pulmonary or cardiac repairs due to trauma. Vascular trauma concentrates the lowest exposure for residents. In this area, the percentage of residents that performed any aortic repairs is 79%, and in other procedures such as arterio-venous fistula and vascular bypass, the number of cases performed by residents is low (only 10% performed at least 5 of each procedure). 

## DISCUSSION

Trauma is associated with a high morbimortality rate, the fast and standardized management with early bleeding control is fundamental^29^ ^,^ ^15^. Patients’ treatment is often performed by general surgeons at the emergency department of general hospitals^4^. Additionally, there is an increasing concern of the quality of some surgical skills acquired during residency training^18^ ^,^ ^22^ ^,^ ^30^ ^,^ ^34^. This justifies the need to investigate the level of trauma management skills of recently graduated surgeons.

To our knowledge, there are no previous local studies assessing this issue; therefore, we performed a descriptive study as a first exploration of the current situation. Moreover, the use of a survey as the assessment tool allows us to cost-effectively increase the sample obtained and, therefore, the results’ representativeness. This approach is useful in countries with a wide variety of training programs.

The results show that, in our country, recently graduated surgeons report a high level of competence to perform procedures of low complexity such as abdominal procedures (bowel anastomosis, colonic repair, and splenectomy). On the contrary, high complexity procedures such as cervical trauma or vascular injuries are the ones with the lowest levels of reported competence. This finding is relevant since hemorrhagic shock is the main cause of potentially avoidable death in severe trauma^15^ ^,^ ^29^. Figure 1 summarizes the results according to each procedure and can be used as a reference for future or comparative studies.

Despite the debate that surrounds the association between the numbers of cases performed by a resident and the acquired competences, in this study, the perception of competences was proportional to the number of procedures performed^27^. The exposure of in-training surgeons to trauma scenarios should be increased in order to improve their competence in the area. Probably, longer periods of residency may be a potential solution to overcome these barriers (such as extending the three-year to a five-year residency).

The low-level of competence and the reduced exposure to trauma cases has also been reported in international literature. A 15-year study in the United States demonstrated a decrease in the number of trauma cases managed by general surgery residents^34^. In Canada, flaws in trauma surgical training have also been detected. At least 20% of their population do not have access to a trauma center with expert trauma surgeons, care is also delivered by general surgeons in local hospitals^9^ ^,^ ^13^ ^,^ ^22^.

Since the decreasing exposure to this kind of case is inevitable, new strategies must be sought and implemented in the training of trauma skills for surgery residents. In other areas of surgery, such as laparoscopic surgery, the training has been complemented with simulation-based programs^17^. These programs have demonstrated to shorten the learning curve and the transfer of the acquired skills to the operating room^6^ ^,^ ^8^ ^,^ ^38^. In trauma surgery, some training courses exists such as the ATLS, widely used in our country, but focuses on general skills rather than the acquisition of surgical competences^24^ ^,^ ^28^. Other courses, like the ATOM, ASSET (ACS), or DQT (from the Panamerican Society of Trauma), enable the acquisition of surgical skills, but they are less available and are often associated with animal ethical concerns and high costs^1^ ^,^ ^19^.

The findings in this work, based on personal reports, reveal an opportunity to improve surgical training programs. This situation must continue to be studied with an objective assessment of surgical skills employing structured tests^37^ ^,^ ^41^.

## CONCLUSION

In summary, recently graduated surgeons in Chile report a high level of surgical competence to treat trauma cases of low and intermediate complexity, but a low level to treat the highly complex ones such as those with vascular compromise or in a cervical location. This must motivate the objective assessment of these skills, and new curricular solutions must be proposed to acquire the missing skills.
